# A Comparison of the Awareness, Attitude, and Uptake of COVID-19, Hepatitis B Virus, and Yellow Fever Vaccines Between Rural and Urban Respondents in Edo State, Nigeria

**DOI:** 10.7759/cureus.44352

**Published:** 2023-08-29

**Authors:** John O Osarenkhoe, Godwin O Agbon, Hendrith Esene, Victor Ohenhen, Aniekeme S Bassey

**Affiliations:** 1 Internal Medicine and Cardiology, Igbinedion University Teaching Hospital, Okada, NGA; 2 Obstetrics and Gynecology, Igbinedion University Teaching Hospital, Okada, NGA; 3 Community Medicine, Igbinedion University Teaching Hospital, Okada, NGA; 4 Obstetric and Gynecology, Central Hospital, Benin, NGA; 5 Radiology, Babcock University Teaching Hospital, Ilishan-Remo, NGA

**Keywords:** side effects, yellow fever, hbv, covid-19, vaccines, vaccine-preventable diseases

## Abstract

Introduction: Despite the target set by WHO, Africa still falls short when it comes to individuals’ use of COVID-19 vaccines. There is a similar pattern of low vaccine usage for the hepatitis B virus (HBV) and yellow fever (YF).

Aim and objective: The objective of our study is to compare the awareness, attitude, and uptake of vaccine-preventable diseases (VPD), COVID-19, HBV, and YF, between a rural and an urban community in Nigeria.

Methodology: The study was a descriptive cross-sectional study carried out between January 2022 and December 2022 in a rural community, Okada, and an urban community, Benin, in Edo State, Nigeria. A total of 283 rural participants and 483 urban participants were interviewed. SPSS Statistics version 26 (IBM Corp. Released 2019. IBM SPSS Statistics for Windows, Version 26.0. Armonk, NY: IBM Corp.) was used for data collection and analysis. The significant value was set at P<0.05.

Results: A major percentage of both populations, 98.3% urban and 90.1% rural, reported being aware of COVID-19 vaccines (indicated by P<0.001). There was a similar pattern with HBV vaccine awareness. However, awareness of YF vaccines was more common in the rural (63.3%) community than in the urban (55.0%) community. A complete dose uptake of COVID-19 vaccines was reported by 7.7% of the rural and 2.2% of the urban respondents. The major reason for the refusal of vaccine uptake was the fear of possible side effects.

Conclusion: The study showed that vaccine uptake for COVID-19, HBV, and YF is low despite seemingly good awareness of these vaccines. The number one reason for any vaccine refusal is the possibility of experiencing side effects.

## Introduction

A vaccine-preventable disease (VPD) is an infectious disease that can be prevented by the uptake of vaccines [1]. The causes of these diseases range from viruses to bacteria. However, most VPDs have viral causes [1,2]. WHO lists a number of infectious diseases that can be prevented by vaccines, including COVID-19, hepatitis B virus (HBV), and yellow fever (YF) [2].

In January 2020, WHO declared COVID-19 a public health emergency one month after the disease broke out in Hubei Province in the People’s Republic of China [3,4]. The spread of COVID-19 was rapid and deadly [5]. The causative agent in COVID-19 is SARS-CoV-2 [5]. The symptoms of those who contracted COVID-19 ranged from asymptomatic to severe acute respiratory distress syndrome [4,5]. Due to the unprecedented spread of the COVID-19 pandemic, there was accelerated development of vaccines to minimize the severe disease and death toll [6]. Vaccines have been shown to reduce the rate of COVID-19, hospital admissions, and deaths. However, by the end of 2022, the vaccine coverage for developing countries was still substantially below the WHO recommendation [7,8]. As of February 2022, the national coverage of COVID-19 vaccination in Nigeria was only 3%, although it significantly increased to 53% by March 2023. However, it still fell short of the WHO recommendation of 70% [7,9,10].

Meanwhile, HBV is a blood-borne viral pathogen that attacks the liver and can be acute or chronic [11,12]. According to WHO, the number of people living with chronic HBV infection amounted to 296 million, and Africa accounted for 23% of the global HBV burden [12,13]. HBV vaccines have been available since the 1980s, and in 2015, WHO launched a program with a target of 90% coverage of HBV vaccines because of the increasing burden of the disease in Africa, despite the increasing effectiveness of the vaccine [14].

Finally, YF is an acute viral hemorrhagic disease transmitted by infected mosquitos. It is endemic in tropical areas of Africa and Central and South America [15]. A YF epidemic was reported in some parts of Nigeria between September 2017 and 2019 in addition to an increasing trend in Oyo State in Nigeria amid low vaccination uptake [16-18]. WHO has recommended vaccination as the most important means of preventing YF [15].

Despite the availability of vaccines for COVID-19, HBV, and YF, the vaccine coverage in Nigeria and Africa as a whole is below the WHO recommendations. Hence, this study seeks to assess the awareness and attitudes toward COVID-19, HBV, and YF vaccines and how they relate to their vaccine uptake.

## Materials and methods

This study was a descriptive cross-sectional study carried out between January 1 and 31, 2022, at Okada (rural) and Benin (urban) in Edo State, Nigeria. With a confidence level of 95%, power of 99%, and effect size of 0.3, the sample size for the prevalence of the study was calculated using GPower 3.1 statistical software (Heinrich-Heine-Universität Düsseldorf, Düsseldorf, Germany). A total of 741 participants were interviewed: 283 were from the rural and 458 from the urban. The ethical committee of Igbinedion University Teaching Hospital granted ethical approval with reference IUTH/R.24/VOL.1/36.

An interviewer-administered structured questionnaire was developed by the researchers in line with the study objectives. Information obtained included sociodemographic characteristics of the study participants and knowledge of infectious diseases, as well as awareness, attitude, and uptake of COVID-19, HBV, and YF vaccines. The participants were randomly selected, and data was collected in hospitals, churches, and shops by five of the researchers. Data collection lasted for 30 days. Contained in the questionnaire were simple questions, a sample of which is presented in the Appendices section.

Knowledge of infectious disease was ascertained if respondents were able to answer correctly about the causative agents of the disease. Awareness of vaccines was ascertained if respondents were simply aware of the availability of vaccines for the said infectious disease. The inclusion criteria were met by every adult ≥18 years of age. Those <18 years were excluded from the study.

We entered and analyzed the data obtained by using SPSS Statistics version 26 (IBM Corp. Released 2019. IBM SPSS Statistics for Windows, Version 26.0. Armonk, NY: IBM Corp.). We presented the data using tables and charts. We also used frequencies and percentages to present categorical variables, and levels of association were tested using a chi-square test. Furthermore, we summarized continuous data using mean and standard deviation. The level of significance was set at P<0.05.

## Results

As shown in Table 1, the sociodemographic distribution of the study participants shows male preponderance among respondents in the rural community (50.5%) and female preponderance among respondents in the urban community (81.0%). The highest number of respondents were enrolled in or had completed tertiary education in both rural (52.7%) and urban (57.6%) communities. Most respondents were single in the rural community (60.1%) and married in the urban community (50.9%). The mean ages (in years) and standard deviation of the communities were 31.44 (±13.96) for the rural and 29.55 (±10.13) for the urban.

**Table 1:** Sociodemographic distribution of the study participants N: total number, n: frequency, %: percentage

Variables	Rural N = 283 n (%)	Urban N = 458 n (%)
Sex		
Male	143 (50.5)	87 (19.0)
Female	140 (49.5)	371 (81.0)
Education		
Primary	31 (11.0)	24 (5.2)
Secondary	75 (26.4)	113 (24.7)
Tertiary	149 (52.7)	264 (57.6)
Postgraduate	20 (7.1)	48 (10.5)
None	8 (2.8)	9 (2.0)
Marital status		
Single	170 (60.1)	225 (49.1)
Married	112 (39.6)	233 (50.9)
Divorced	1 (0.4)	0 (0.0)

As shown in Table 2, 99.1% of respondents in the urban community compared to 90.8% of respondents in the rural community have knowledge of infectious diseases (indicated by P<0.001). We noted the awareness of COVID-19 between the urban (99.1%) and rural (92.1%) communities that were statistically significant (indicated by P<0.001). A statistically significant result was also noted regarding the awareness of HBV in the urban (98.9%) and rural (84.5%) communities (indicated by P<0.001). More respondents in the rural community (77.0%) than in the urban community (70.5%) were aware of YF. However, the difference was not statistically significant (indicated by P<0.058).

**Table 2:** Awareness of infectious diseases: COVID-19, HBV, and YF N: total number, n: frequency, %: percentage, X2: chi-square test statistics, *: significant P-value, HBV: hepatitis B virus, YF: yellow fever

Variables	Rural N = 283 n (%)	Urban N = 458 n (%)	X^2^	p-value
Knowledge of infectious disease	257 (90.8)	454 (99.1)	31.12	<0.001*
Knowledge of COVID-19	263 (92.9)	454 (99.1)	21.41	<0.001*
Knowledge of HBV	239 (84.5)	453 (98.9)	58.19	<0.001*
Knowledge of YF	217 (77.0)	323 (70.5)	3.66	0.058

As shown in Table 3, there is more awareness of COVID-19 and HBV vaccines in the urban community compared to the rural community (indicated by P<0.001). However, the awareness of YF vaccines is higher in the rural community (63.3%) compared to the urban community (55.0%) (indicated by P=0.027). No statistically significant difference was noticed in the uptake of COVID-19 vaccines between the rural (28.3%) and urban (30.1%) communities (indicated by P=0.589). Similarly, no statistically significant difference was seen in the uptake of HBV vaccines between the rural (25.1%) and urban (31.7%) communities (indicated by P=0.056). However, the uptake of YF vaccines was higher in the urban community (48.9%) than in the rural community (33.2%) (indicated by P<0.001).

**Table 3:** Awareness and uptake of COVID-19, HBV, and YF vaccines N: total number, n: frequency, %: percentage, X2: chi-square test statistics, *: significant p-value, HBV: hepatitis B virus, YF: yellow fever

Variables	Rural N = 283 n (%)	Urban N = 458 n (%)	X^2^	p-value
Awareness of COVID-19 vaccines	255 (90.1)	450 (98.3)	25.12	<0.001*
Awareness of HBV vaccines	223 (78.8)	431 (94.1)	58.19	<0.001*
Awareness of YF vaccines	179 (63.3	252 (55.0)	4.87	0.027*
Uptake of COVID-19 vaccines	80 (28.3)	138 (30.1)	0.292	0.589
Uptake of HBV vaccines	71 (25.1)	145 (31.7)	3.66	0.056
Uptake of YF vaccines	94 (33.2)	224 (48.9)	17.58	<0.001*

As shown in Table 4, more rural respondents, 7.1%, had up to three doses of COVID-19 vaccine compared to the urban respondents at 2.2%. However, most respondents stopped at their second dose in both the rural (11.3%) and urban (14.0%) communities (indicated by P=0.002). There was no statistically significant difference in the three-dose uptake of HBV between the rural (9.2%) and urban (10.7%) communities (indicated by P=0.231). Most respondents who had YF vaccines had up to three doses in both the rural (14.8%) and urban (33.0%) communities, and the difference was statistically significant between them (indicated by P<0.001).

**Table 4:** COVID-19, HBV, and YF vaccine uptake doses N: total number, n: frequency, %: percentage, X2: chi-square test statistics, *: significant p-value, HBV: hepatitis B virus, YF: yellow fever

Variables	Rural N = 283 n (%)	Urban N = 458 n (%)	X^2^	p-value
COVID-19 vaccine uptake doses				
One dose	26 (9.2)	63 (13.8)	14.44	0.002*
Two doses	32 (11.3)	54 (14.0)		
Three doses	20 (7.1)	10 (2.2)		
HBV vaccine uptake doses				
One dose	22 (7.8)	49 (10.7)	4.302	0.231
Two doses	22 (7.8)	47 (10.3)		
Three doses	26 (9.2)	49 (10.7)		
YF vaccine uptake doses				
One dose	30 (10.6)	34 (7.4)	38.37	<0.001*
Two doses	10 (3.5)	33 (7.2)		
Three doses	42 (14.8)	151 (33.0)		

**Figure 1:**
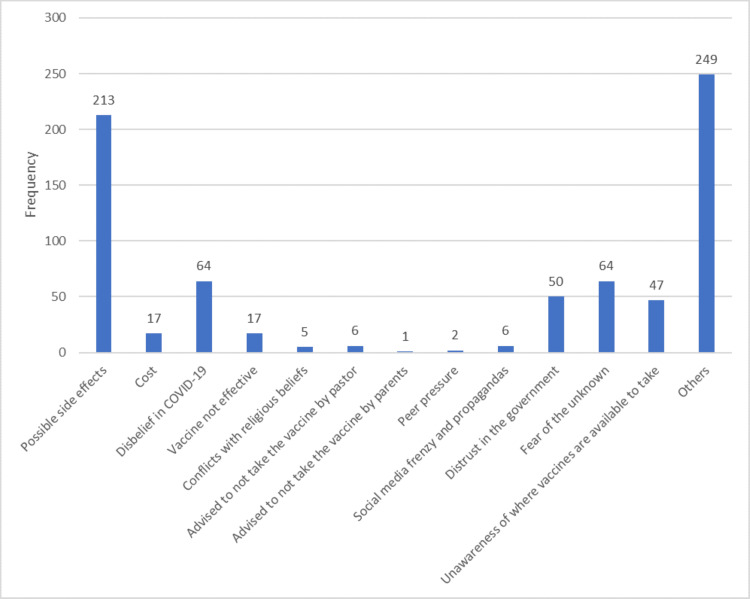
Bar chart showing the reasons for not taking COVID-19 vaccines

Figure 1 shows that the major reason given by respondents for not taking COVID-19 vaccines was possible side effects at 28.7%. Other major reasons were fear of the unknown (8.6%) and disbelief in COVID-19 (8.64%).

**Figure 2:**
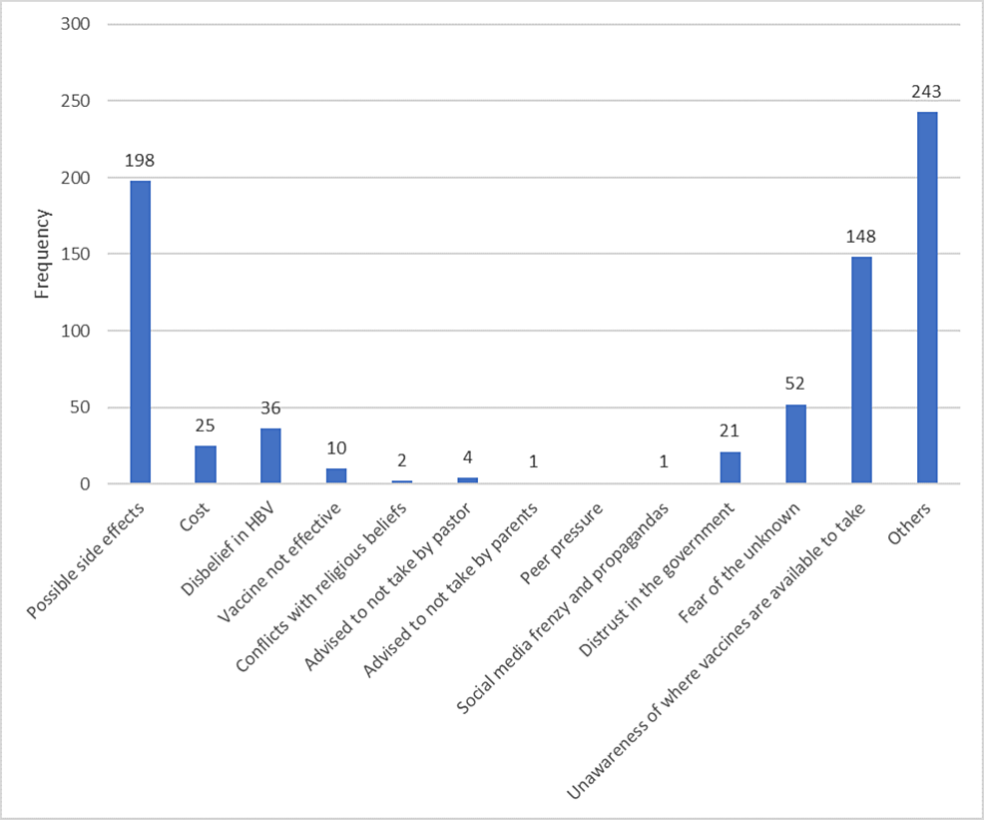
Bar chart showing the reasons for not taking HBV vaccines

Figure 2 shows that the major reason given by respondents (26.7%) for not taking HBV vaccines was possible side effects. Another major reason was unawareness of where the vaccine was available.

**Figure 3:**
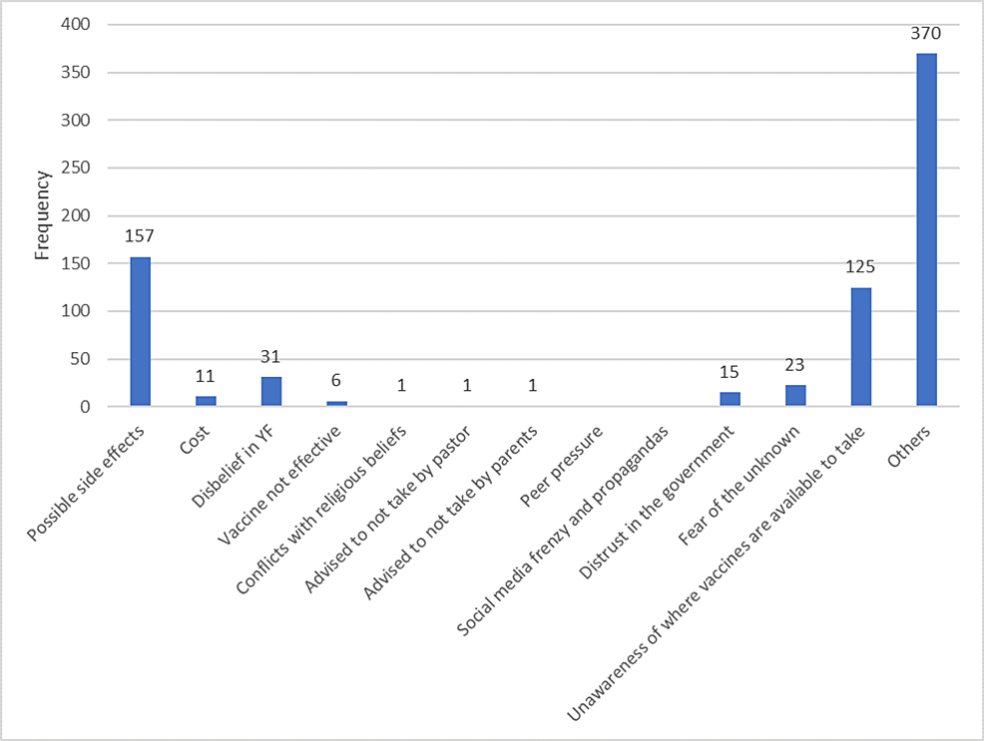
Bar chart showing the reasons for not taking HBV vaccines

Figure 3 shows that the major reason, at 21.2%, given by respondents for not taking YF vaccines was possible side effects. Another major reason was unawareness of where the vaccine was available.

## Discussion

The findings in our study showed that there is a seemingly high awareness of infectious diseases, COVID-19 and HBV. The urban respondents express more knowledge of infectious diseases, COVID-19 and HBV, than the rural respondents. In contrast, there is more knowledge of YF in the rural respondents than in the urban respondents. A study conducted in North Central Nigeria and published in 2020 showed that 99.5% of the population had good knowledge of COVID-19, similar to the result we obtained from our urban respondents [19]. However, studies conducted in Syria, Ethiopia. and Indonesia showed relatively lower knowledge of COVID-19 at 88%, 74%, and 70%, respectively [20-22]. A study conducted in South West Nigeria showed that 53.9% of the respondents had sufficient knowledge of HBV [23].

Awareness of COVID-19 vaccines was significantly greater among the urban respondents (98.3%) compared to the rural respondents (90.1%). Interestingly, there was no statistically significant difference between the uptake of COVID-19 vaccines between the urban (30.1%) and rural (28.3%) respondents. There was an even lower uptake of the third dose in the urban (2.2%) and rural (7.1%) communities. Although there is little published data that shows public awareness of COVID-19 vaccines, a study conducted among some university students in Nigeria did show knowledge of the different COVID-19 vaccines that were available [24]. As of May 2023, WHO reports that 5.9% of Nigerians have received up to the third booster dose of COVID-19 vaccines [25].

Meanwhile, awareness of HBV vaccines was higher in the urban respondents (94.1%) than in the rural respondents (78.8%). The seemingly high awareness level of HBV vaccines did not translate to the uptake of these vaccines, as vaccine uptake was quite low in the urban (10.9%) and rural (9.2%) respondents.

Finally, YF vaccine awareness was higher among the rural respondents (63.3%) compared to the urban respondents (55.0%). Despite that difference, the uptake of YF vaccines was higher among the urban respondents (48.9%) compared to the rural respondents (33.1%). Thirty-three percent of those who took YF vaccines among the urban respondents continued to complete the dose compared to 14.8% of the rural respondents. It does seem that there is general acceptance of YF vaccines among respondents compared to COVID-19 and HBV vaccines.

The respondents who did not take COVID-19, HBV, and YF vaccines listed the possibility of side effects as the major reason for not doing so. This was comparable to a study done in Abuja that showed 52.9% of vaccine hesitancy was due to fear of side effects [26]. Another major barrier to vaccine uptake in the cases of HBV and YF was unawareness of where to get the vaccine.

This study compared the differences in awareness, attitude, and uptake of COVID-19, HBV, and YF vaccines between the rural and urban respondents in one state. A study with a larger sample size that covers more regions and states in Nigeria would have been more beneficial in ascertaining the differences in the parameters being compared. There were also language limitations between the respondents (especially in the rural) and researchers.

## Conclusions

Our study showed that the awareness of COVID-19 and HBV vaccines is relatively high, although there is still a lag in rural areas, especially regarding HBV vaccines. The awareness of YF vaccines is, however, low among rural and urban respondents. Despite seemingly fair-to-good knowledge of these vaccines, the uptake of these vaccines is low. Furthermore, the difference in awareness level between rural and urban respondents does not translate to the difference between vaccine uptake between rural and urban respondents; hence, the awareness gap does not explain the poor attitude toward vaccine uptake. Fear of possible side effects was the leading contributing factor to the respondents' poor attitude toward vaccine uptake.

## Appendices

Below is a sample of the simple questions contained in the questionnaire: 

1. Sociodemography

a. Write Age (in years) - _____

b. Sex (tick one) - 1. Male 2. Female

c. Education level (tick one) - 1. Primary 2. Secondary 3. Tertiary 4. Post-graduate 5. None

d. Marital status (tick one) - 1. Single 2. Married 3. Divorced

2. Knowledge of infectious diseases

a. Do you know of infectious diseases (tick one)? - 1. Yes 2. No

b. If yes to 2a above, name one infectious disease - _____

3. Awareness of COVID-19, HBV, and YF

a. Have you heard of COVID-19 (tick one)? - 1. Yes 2. No

b. If yes to 3a above, is it infectious (tick one)? - 1. Yes 2. No

4. Awareness and uptake of COVID-19 vaccines

a. Are you aware of COVID-19 vaccines (tick one)? - 1. Yes 2. No

b. If yes to 4a above, have you taken any (tick one)? - 1. Yes 2. No

c. If yes to 4b above, how many doses (tick one)? - 1. One dose 2. Two doses 3. Three doses 4. More than three doses

d. If no to question 4b above, what is the reason for not taking (tick as applicable)? - 1. Possible side effects 2. Cost 3. Disbelief in COVID-19 4. Vaccine not effective 5. Conflict with religious beliefs 6. Advised to not take vaccine by a pastor 7. Advised to not take the vaccine by parents 8. Peer pressure 9. Social media frenzy and propaganda 10. Distrust in government 11. Fear of the unknown 12. Unawareness of where vaccines are available to take 13. Others _____
